# Supplementary material for: Concerted SUMO-targeted ubiquitin ligase activities of TOPORS and RNF4 are essential for stress management and cell proliferation
Source: Nat Struct Mol Biol. 2024 Apr 22;31(9):1355–67. doi: 10.1038/s41594-024-01294-7 (PMC11402782; doi:10.1038/s41594-024-01294-7)
Supplement: Supplementary file 1 — Supplementary Figs. 1 and 2, legends for Supplementary Data 1–4, Supplementary Methods and references for Supplementary Information [file 41594_2024_1294_MOESM1_ESM.pdf]

nature structural & molecular biology

Article https://doi.org/10.1038/s41594-024-01294-7

Concerted SUMO-targeted ubiquitin ligase activities of TOPORS and RNF4 are essential for stress management and cell proliferation

In the format provided by the authors and unedited

Contents

Supplementary Figures 1 and 2 with legends

Supplementary Methods

References for Supplementary Information

Supplementary Figures

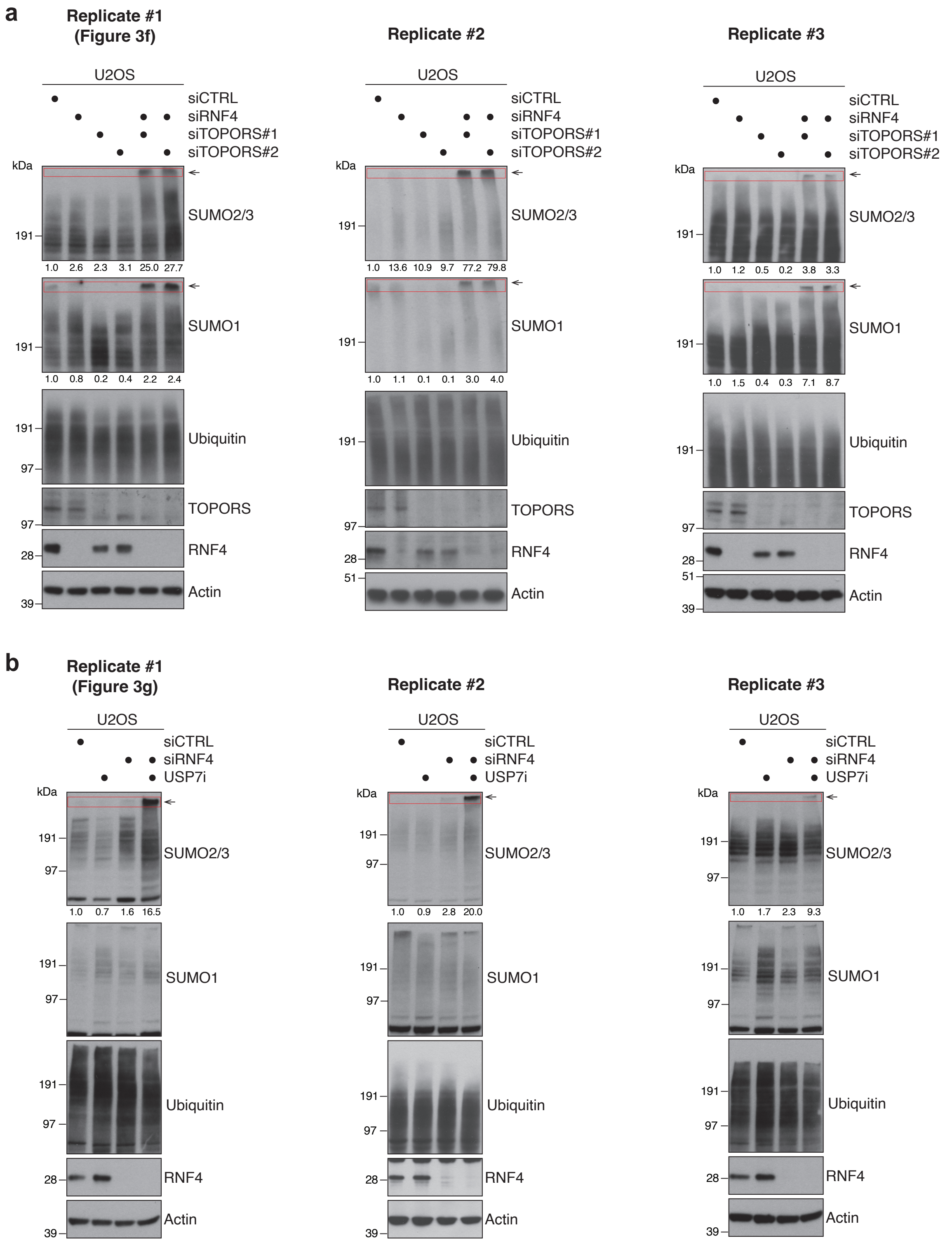

Supplementary Figure 1.
a. Independent biological replicates and quantification of data in **Figure 3f**. Boxed areas (red) were quantified. **b.** Independent biological replicates and quantification of data in **Figure 3g**. Boxed areas (red) were quantified.

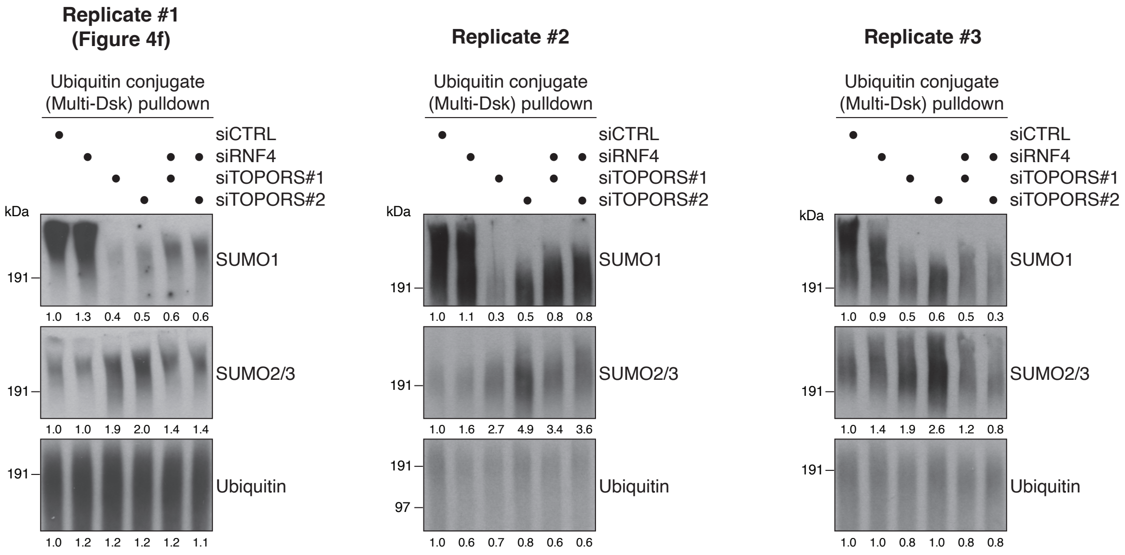

Supplementary Figure 2.
Independent biological replicates and quantification of data in **Figure 4e**.

Supplementary Methods

Mass spectrometry analysis of DNMT1 pulldowns
U2OS cells transfected with siRNAs for 72 h were synchronized by single-round thymidine treatment. Cells were released from synchronization and treated or not with 5-AzadC for 30 min in early S phase. Cells were then collected and lysed in denaturing buffer (20 mM Tris, pH 7.5; 50 mM NaCl; 1 mM EDTA; 0.5% NP40; 0.5% SDS, 0.5% sodium deoxycholate; 1 mM DTT) and subjected to IP using DNMT1-trap beads, as described above. After overnight incubation, beads were washed extensively in denaturing buffer. To make samples MS-compatible, beads were washed with minimal washing buffer (50 mM Tris, pH 7.5; 150 mM NaCl) for 15 min and transferred to Protein LoBind tubes (Eppendorf). The washing procedure was repeated twice and beads were again transferred to new Protein LoBind tubes. Beads were then washed one final time with minimal washing buffer (at pH 8.0) for 15 min. Digestion of proteins was performed on-beads, using 250 ng (5 ng/µl) trypsin, with 1 h pre-incubation on ice and overnight incubation with shaking at 30 °C. Digests were cleared by centrifugation through 0.45 µm spin filters, after which peptides were reduced and alkylated using chloroacetamide and tris(2-carboxyethyl)phosphine at final concentrations of 5 mM. Peptides were purified on StageTips at high pH, with C18 StageTips prepared in-house, by layering four plugs of C18 material (Sigma-Aldrich, Empore SPE Disks, C18, 47 mm) per StageTip. Activation of StageTips was performed with 100 µL 100% methanol, followed by equilibration using 100 µL 80% acetonitrile (ACN) in 200 mM ammonium hydroxide, and two washes with 100 µL 50 mM ammonium hydroxide. Samples were basified to pH >10 by addition of one fifth volume of 200 mM ammonium hydroxide, after which they were loaded on StageTips. Subsequently, StageTips were washed twice using 100 µL 50 mM ammonium hydroxide, after which peptides were eluted using 80 µL 25% ACN in 50 mM ammonium hydroxide. All fractions were dried to completion using a SpeedVac at 60 °C. Dried peptides were dissolved in 11 µL 0.1% formic acid and stored at −20 °C until analysis using mass spectrometry (MS).

All samples were analyzed on an EASY-nLC 1200 system (Thermo) coupled to an Orbitrap Exploris 480 mass spectrometer (Thermo). Samples were analyzed on 20 cm long analytical columns, with an internal diameter of 75 µm, and packed in-house using ReproSil-Pur 120 C18-AQ 1.9 µm beads (Dr. Maisch). The analytical column was heated to 40 °C, and elution of peptides from the column was achieved by application of gradients with stationary phase Buffer A (0.1% formic acid (FA)) and increasing amounts of mobile phase Buffer B (80% ACN in 0.1% FA). The primary analytical gradient ranged from 5 %B to 32 %B over 60 min, followed by a tail-end increase to 42 %B over 5 min to ensure full peptide elution, followed by a washing block of 15 min. Ionization was achieved using a NanoSpray Flex NG ion source (Thermo), with spray voltage set at 2 kV, ion transfer tube temperature to 275 °C, and RF funnel level to 40%. Full scan range was set to 300-1,300 m/z, MS1 resolution to 120,000, MS1 AGC target to "200" (2,000,000 charges), and MS1 maximum injection time to "Auto". Precursors with charges 2-6 were selected for fragmentation using an isolation width of 1.3 m/z and fragmented using higher-energy collision disassociation (HCD) with normalized collision energy of 25. Monoisotopic Precursor Selection (MIPS) was enabled in "Peptide" mode. Precursors were prevented from being repeatedly sequenced by setting expected peak width to 40 s, and setting dynamic exclusion duration to 80 s, with an exclusion mass tolerance of 15 ppm, exclusion of isotopes, and exclusion of alternate charge states for the same precursor. MS/MS resolution was set to 45,000, MS/MS AGC target to "200" (200,000 charges), MS/MS intensity threshold to 230,000, MS/MS maximum injection time to "Auto", and TopN to 9.

Mass spectrometry data analysis (DNMT1 pulldowns)

All RAW files were analyzed using MaxQuant software (version 1.5.3.30) [1,2]. Default MaxQuant settings were used, with exceptions outlined below. For generation of the theoretical spectral library, the HUMAN.FASTA database was extracted from UniProt on 3 September, 2021. Protein N-terminal acetylation (default), methionine oxidation (default), and lysine ubiquitylation (i.e. GlyGly), were included as potential variable modifications, with a maximum allowance of 3 variable modifications per peptide. First search mass tolerance was set to 10 ppm, and maximum charge state of considered precursors to 6. Label-free quantification (LFQ) was enabled, and "Fast LFQ" was disabled. Second peptide search was enabled (default) and matching between runs was enabled with a match time window of 1 min and an alignment time window of 30 min. Note that the alignment time window was increased (from default 20 min) because during one run ("20221116_EXPL7_LC6_IAH_collab_JL_D-IP_siD_AZA_01.raw") contact closure initiated early, resulting in the mass spectrometer starting the recording during sample loading, causing all peaks in the gradient to be shifted by ~15 min. Minimum Score and minimum Delta Score for modified peptides were increased to 80 and 40, respectively, and site decoy fraction was set to 0.02. Data were filtered by posterior error probability to achieve a false discovery rate of <1% (default), at both the peptide-spectrum match and the protein assignment levels.

MS data statistics (DNMT1 pulldowns)

Statistical handling of MS data was performed using Perseus software (versions 1.5.5.3 and 1.6.14.0) [3], and visualized using GraphPad Prism (version 9.5.1). MS data was filtered by removing reverse-database hits and potential contaminants. Following 2log transformation, only proteins and peptides observed at n=4/4 in at least one experimental condition were considered. In case of protein-level quantification, missing values were globally imputed (down shift 1.8, width 0.3).

Proteomic analysis of PML body components

The generation of U2OS PML-KO/YFP-PML cells and purification of PML bodies has been described previously [4]. The purification procedure here was followed exactly as detailed,

except that cells were grown in SILAC medium for at least 8 doublings prior to expansion from a single 75 cm^2 flask to five confluent 15 cm dishes. Cells were treated with 1 μM arsenic trioxide for 2 h (Heavy condition) or left untreated (Light condition). Purified PML body samples from each SILAC condition were mixed in 1:1 ratio by protein mass, then fractionated by SDS-PAGE and the lane cut into 7 slices. Tryptic peptides were extracted and approximately 15-30% of the total peptide yield for each slice was analyzed by LC-MS/MS using a 150-min elution gradient and a top 10 data-dependent method on a Q Exactive mass spectrometer broadly as described previously [4]. The remaining tryptic peptides were further digested with GluC and approximately half of the yield was analyzed by LC-MS/MS using a 180-min elution gradient and similar top 10 data-dependent mass spectrometer settings. MS data were processed in MaxQuant version 1.6.1.0 [5,6] using a UniProt human database containing 73,920 proteins downloaded in 2019 and the YFP-PML sequence. Oxidized methionine and acetylated protein N-termini were selected as variable modifications and carbamidomethyl-C was a fixed modification. Digestion assumed Trypsin/P (maximum missed cleavages of 2) or Trypsin/P plus GluC (maximum missed cleavages of 5) depending on the peptide preparations analyzed. Match between runs and advanced ratio estimation were selected. Using 1% FDR filtering against a reverse decoy database at peptide and protein levels, data for 1,772 protein groups were returned which was reduced to 1,025 by removal of decoy proteins, proteins only identified by modified peptides, proteins from the contaminants database, proteins without a reported H/L ratio and proteins with fewer than three unique peptides.

References for Supplementary Information

1. Cox, J. & Mann, M. MaxQuant enables high peptide identification rates, individualized p.p.b.-range mass accuracies and proteome-wide protein quantification. *Nat. Biotechnol* **26**, 1367-1372 (2008).
2. Cox, J. et al. Andromeda: a peptide search engine integrated into the MaxQuant environment. *J. Proteome. Res* **10**, 1794-1805 (2011).
3. Tyanova, S. et al. The Perseus computational platform for comprehensive analysis of (prote)omics data. *Nat Methods* **13**, 731-40 (2016).
4. Jaffray, E.G. et al. The p97/VCP segregase is essential for arsenic-induced degradation of PML and PML-RARA. *J Cell Biol* **222**(2023).
5. Cox, J. & Mann, M. MaxQuant enables high peptide identification rates, individualized p.p.b.-range mass accuracies and proteome-wide protein quantification. *Nat Biotechnol* **26**, 1367-72 (2008).
6. Cox, J. et al. Andromeda: a peptide search engine integrated into the MaxQuant environment. *J Proteome Res* **10**, 1794-805 (2011).
