# Supplementary material for: Concerted SUMO-targeted ubiquitin ligase activities of TOPORS and RNF4 are essential for stress management and cell proliferation
Source: Nat Struct Mol Biol. 2024 Apr 22;31(9):1355–67. doi: 10.1038/s41594-024-01294-7 (PMC11402782; doi:10.1038/s41594-024-01294-7)
Supplement: Supplementary file 2 — Reporting Summary [file 41594_2024_1294_MOESM2_ESM.pdf]

nature portfolio

Corresponding author(s): Niels Mailand

Last updated by author(s): Mar 4, 2024

Reporting Summary

Nature Portfolio wishes to improve the reproducibility of the work that we publish. This form provides structure for consistency and transparency in reporting. For further information on Nature Portfolio policies, see our Editorial Policies and the Editorial Policy Checklist.

Statistics

For all statistical analyses, confirm that the following items are present in the figure legend, table legend, main text, or Methods section.

n/a	Confirmed	
☐	☒	The exact sample size (*n*) for each experimental group/condition, given as a discrete number and unit of measurement
☐	☒	A statement on whether measurements were taken from distinct samples or whether the same sample was measured repeatedly
☐	☒	The statistical test(s) used AND whether they are one- or two-sided<br>*Only common tests should be described solely by name; describe more complex techniques in the Methods section.*
☒	☐	A description of all covariates tested
☐	☒	A description of any assumptions or corrections, such as tests of normality and adjustment for multiple comparisons
☐	☒	A full description of the statistical parameters including central tendency (e.g. means) or other basic estimates (e.g. regression coefficient) AND variation (e.g. standard deviation) or associated estimates of uncertainty (e.g. confidence intervals)
☐	☒	For null hypothesis testing, the test statistic (e.g. *F*, *t*, *r*) with confidence intervals, effect sizes, degrees of freedom and *P* value noted<br>*Give P values as exact values whenever suitable.*
☒	☐	For Bayesian analysis, information on the choice of priors and Markov chain Monte Carlo settings
☒	☐	For hierarchical and complex designs, identification of the appropriate level for tests and full reporting of outcomes
☒	☐	Estimates of effect sizes (e.g. Cohen's *d*, Pearson's *r*), indicating how they were calculated

Our web collection on statistics for biologists contains articles on many of the points above.

Software and code

Policy information about availability of computer code

Data collection	Incucyte S3 Live-Cell Analysis System (version 2021A), SoftWoRx software (version 7.0.0; GE Healthcare), FlowJo (version 10.8.1).
Data analysis	ScanR analysis software (version 2.8.1; Olympus), Spotfire (version 10.5.0; Tibco), SoftWoRx software (version 7.0.0; GE Healthcare), FlowJo, GraphPad Prism (version 9.5.1), MaxQuant (versions 1.5.3.30 and 1.6.1.0), Perseus (versions 1.5.5.3 and 1.6.14.0). Aanalysis pipeline for gene-trap insertion orientation bias (sense-ratio) can be found at GitHub (https://github.com/BrummelkampResearch/phenosaurus).

For manuscripts utilizing custom algorithms or software that are central to the research but not yet described in published literature, software must be made available to editors and reviewers. We strongly encourage code deposition in a community repository (e.g. GitHub). See the Nature Portfolio guidelines for submitting code & software for further information.

Data

Policy information about availability of data

All manuscripts must include a data availability statement. This statement should provide the following information, where applicable:

- Accession codes, unique identifiers, or web links for publicly available datasets
- A description of any restrictions on data availability
- For clinical datasets or third party data, please ensure that the statement adheres to our policy

The mass spectrometry proteomics data have been deposited to the ProteomeXchange Consortium (ref. 60) via the Proteomics Identification (PRIDE) partner repository (http://www.ebi.ac.uk/pride) under dataset ID PXD041717 (https://proteomecentral.proteomexchange.org/cgi/GetDataset?ID= PXD041717)

(Supplementary Data 2) and dataset ID PXD041718 (https://proteomecentral.proteomexchange.org/cgi/GetDataset?ID= PXD041718) (Supplementary Data 3). Raw sequencing data from genetic screens have been deposited to the NCBI Sequence Read Archive (https://www.ncbi.nlm.nih.gov/sra) under dataset ID PRJNA975887 (https://dataview.ncbi.nlm.nih.gov/object/PRJNA975887?reviewer=af0jv5dqrcq2pd277kfdb5lk7e). All other data supporting the findings of this study are available within the article and supplementary information. Source data are provided with this paper.

Research involving human participants, their data, or biological material

Policy information about studies with human participants or human data. See also policy information about sex, gender (identity/presentation), and sexual orientation and race, ethnicity and racism.

Reporting on sex and gender	N/A
Reporting on race, ethnicity, or other socially relevant groupings	N/A
Population characteristics	N/A
Recruitment	N/A
Ethics oversight	N/A

Note that full information on the approval of the study protocol must also be provided in the manuscript.

Field-specific reporting

Please select the one below that is the best fit for your research. If you are not sure, read the appropriate sections before making your selection.

☒ Life sciences ☐ Behavioural & social sciences ☐ Ecological, evolutionary & environmental sciences

For a reference copy of the document with all sections, see nature.com/documents/nr-reporting-summary-flat.pdf

Life sciences study design

All studies must disclose on these points even when the disclosure is negative.

Sample size	Sample sizes were based on previously reported data. No statistical method was used to predetermine sample size.
Data exclusions	No data were excluded from the analysis.
Replication	All experimental findings shown in this study were independently replicated at least twice with similar outcome. Information about replication is provided in the figure legends.
Randomization	The samples were not randomized. Randomization is generally not relevant for this study since we are working with cell populations and not test subjects.
Blinding	The investigators were not blinded to group allocation during data collection and analysis, but great care was taken to avoid bias.

Reporting for specific materials, systems and methods

We require information from authors about some types of materials, experimental systems and methods used in many studies. Here, indicate whether each material, system or method listed is relevant to your study. If you are not sure if a list item applies to your research, read the appropriate section before selecting a response.

Materials & experimental systems

n/a	Involved in the study	
☐	☒	Antibodies
☐	☒	Eukaryotic cell lines
☒	☐	Palaeontology and archaeology
☒	☐	Animals and other organisms
☒	☐	Clinical data
☒	☐	Dual use research of concern
☒	☐	Plants

Methods

n/a	Involved in the study	
☒	☐	ChIP-seq
☐	☒	Flow cytometry
☒	☐	MRI-based neuroimaging

Antibodies

Antibodies used	Antibodies to human proteins used in this study included: Actin (1:20,000 dilution (MAB1501 (clone ID: C4), Millipore, RRID:AB_2223041)); DNMT1 (1:1,000, described in ref. 11); FLAG (1:1,000 (A00187 (clone ID: 5A8E5), GenScript, RRID:AB_1720813)); GFP (1:5,000, Abcam (ab6556, RRID:AB_305564)); HA (1:1,000 (11867423001 (clone ID: 3F10), Roche, RRID:AB_390918)); Histone H3 (1:20,000, Abcam (ab1791, RRID:AB_302613)); PARP1 (1:1,000, Santa Cruz Biotechnology (sc-8007 (clone ID: F2), RRID:AB_628105)); PIAS1 (1:1,000 (ab77231, Abcam, RRID:AB_1524188)); RNF4 (1:5,000, described in ref. 54); SUMO1 (1:1,000, Thermo Fisher Scientific (33-2400 (clone ID: 21C7), RRID:AB_2533109)); SUMO2/3 (1:1,000, Abcam (ab3742, RRID:AB_304041); 1:1,000, Abcam (ab81371 (clone 1D: 8A2), RRID:AB_1658424)); TOP1 (1:500, Bethyl (A302-590A, RRID:AB_2034875)); TOPORS (1:250, sheep polyclonal raised against full-length human TOPORS); ubiquitin (1:1,000, Santa Cruz Biotechnology (sc-8017 AC (clone ID: P4D1), RRID:AB_2762364); 1:1,000, Millipore (04-263 (clone ID: FK2), RRID:AB_612093)); ubiquitin (K48-linked) (1:1,000, Millipore (05-1307 (clone ID: Apu2), RRID:AB_1587578)); ubiquitin (K63-linked) (1:1,000, (05-1308 (clone ID: Apu3), RRID:AB_1587580)); UBE2K (1:1,000, Cell Signaling Technology (3847, RRID:AB_2210768)); and USP7 (1:1,000, Bethyl (A300-033A, RRID:AB_203276)).
Validation	The specificity of antibodies against TOPORS, RNF4, UBE2K, FLAG, HA, SUMO1 and SUMO2/3 (ab3742) was validated using appropriate knockdown/knockout controls in this study (as shown in the manuscript). The following antibodies were validated in our previous studies: DNMT1 (PMID: 30914427), GFP (PMID: 34346517), SUMO2/3 (ab81371) (PMID: 34346517), ubiquitin (sc-8017 and 04-263) (PMID: 35349166). The following antibodies are commonly used loading controls: Actin, Histone H3. Other antibodies (PARP1, TOP1, ubiquitin (K48-linked), ubiquitin (K63-linked), USP7) were used based on previous validation in the literature and/or manufacturer websites.

Eukaryotic cell lines

Policy information about cell lines and Sex and Gender in Research

Cell line source(s)	Human HeLa (catalog no. CCL-2) and U2OS (catalog no. HTB-96) cells were obtained from ATCC. HEK293-EBNA1-6E cells were a kind gift from Yves Durocher (National Research Council Canada, Montreal, Canada). The generation of HAP1 cells is described in ref. 53.
Authentication	The cell lines were not authenticated.
Mycoplasma contamination	All cell lines used in this study were regularly tested negative for mycoplasma infection.
Commonly misidentified lines (See ICLAC register)	Cell lines used in this study are not included in the ICLAC register of commonly misidentified cell lines.

Flow Cytometry

Plots

Confirm that:

☒ The axis labels state the marker and fluorochrome used (e.g. CD4-FITC).

☒ The axis scales are clearly visible. Include numbers along axes only for bottom left plot of group (a 'group' is an analysis of identical markers).

☒ All plots are contour plots with outliers or pseudocolor plots.

☒ A numerical value for number of cells or percentage (with statistics) is provided.

Methodology

Sample preparation	Cells were collected by trypsinization, fixed in 4% paraformaldehyde in PBS for 15 min and permeabilized in 0.2% Triton-X, 2 FBS in PBS for 20 min at room temperature. Permeabilized cells were washed in FACS buffer (PBS+10% FBS) and incubated with sheep polyclonal anti-DNMT1 antibody 14 diluted in FACS buffer for 90 min at room temperature. Following two washes in FACS buffer, cells were incubated with anti-sheep AlexaFluor 488 (Invitrogen) diluted in FACS buffer for 1 h at room temperature. For EdU co-staining, washed cells were subsequently stained using the Click-iT Plus EdU Alexa Fluor 647 Kit (Invitrogen) according to the manufacturer’s instructions. Following two additional washes in FACS buffer, cells were resuspended in FACS buffer containing 1 µg/ml DAPI (Thermo Fisher) and strained to a single cell solution (40 µm filter).
Instrument	Stained cells were analyzed using a BD LSRFortessa flow cytometer (BD Biosciences) or sorted using a BD FACSAria Fusion.
Software	Analysis was performed with and plots were generated using FlowJo software.
Cell population abundance	Single cell haploid S-phase population based on DAPI/EdU was approximately 35%, of these the top and bottom 5% based on DNMT1 signal were sorted. 13E6 cells were sorted for each channel.
Gating strategy	Cells were selected based on FSC/SSC, single cells gated based on DAPI-A vs DAPI-H, haploid S-phase selected based on EdU signal vs DAPI, DNMT1 high and low based on DNMT1 vs DAPI (Extended Data Figure 1A).

☒ Tick this box to confirm that a figure exemplifying the gating strategy is provided in the Supplementary Information.
