# Supplementary material for: Concerted SUMO-targeted ubiquitin ligase activities of TOPORS and RNF4 are essential for stress management and cell proliferation
Source: Nat Struct Mol Biol. 2024 Apr 22;31(9):1355–67. doi: 10.1038/s41594-024-01294-7 (PMC11402782; doi:10.1038/s41594-024-01294-7)

Source data Supplementary Figure 1 - Uncropped scans

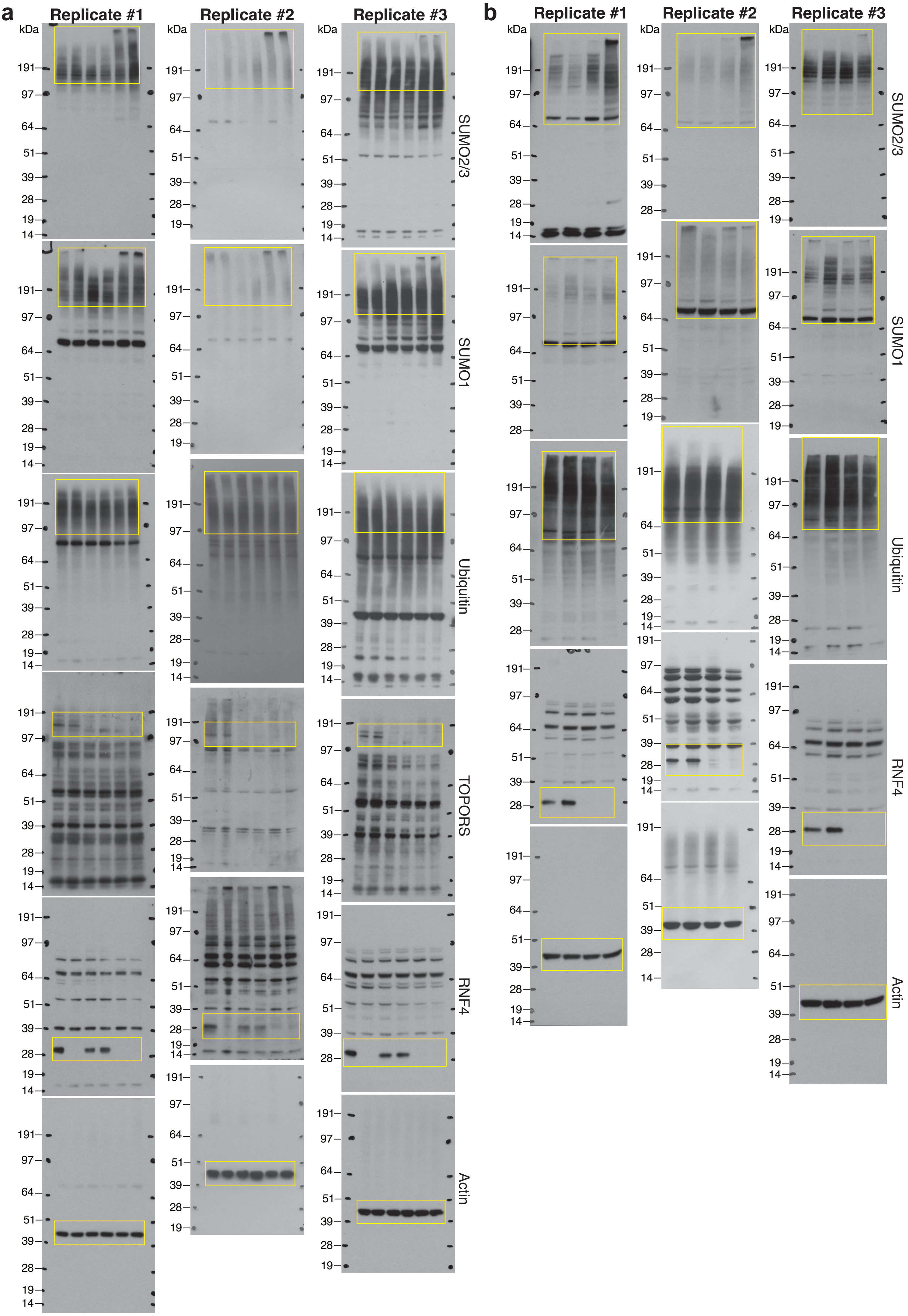

Source data Supplementary Figure 2 - Uncropped scans

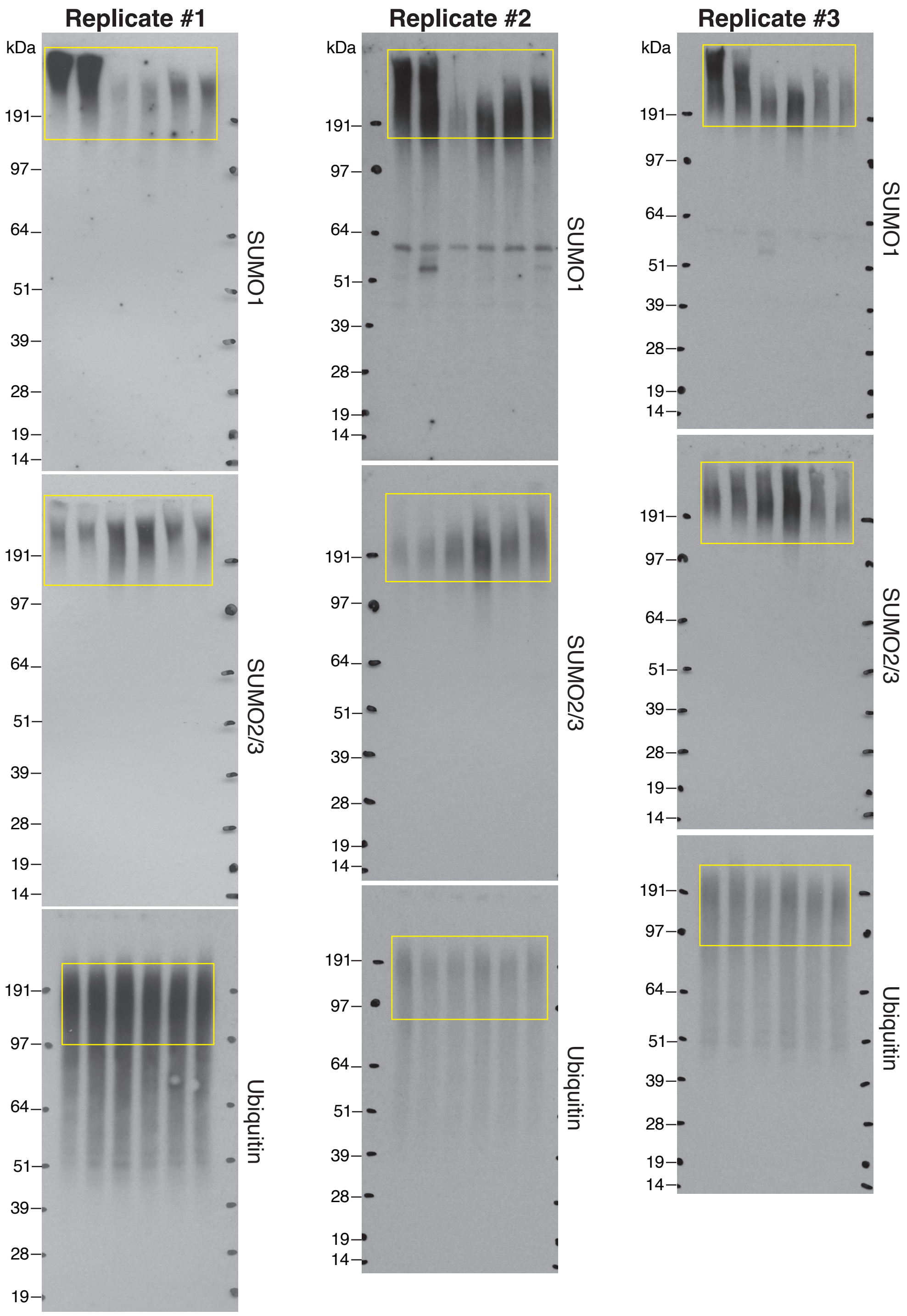

Supplement: Supplementary file 5 — Uncropped western blots for Supplementary Figs. 1 and 2. [file 41594_2024_1294_MOESM5_ESM.pdf]
