# Supplementary figures and images for: Concerted SUMO-targeted ubiquitin ligase activities of TOPORS and RNF4 are essential for stress management and cell proliferation
Source: Nat Struct Mol Biol. 2024 Apr 22;31(9):1355–67. doi: 10.1038/s41594-024-01294-7 (PMC11402782; doi:10.1038/s41594-024-01294-7)

Source Data Figure 1 - Uncropped scans

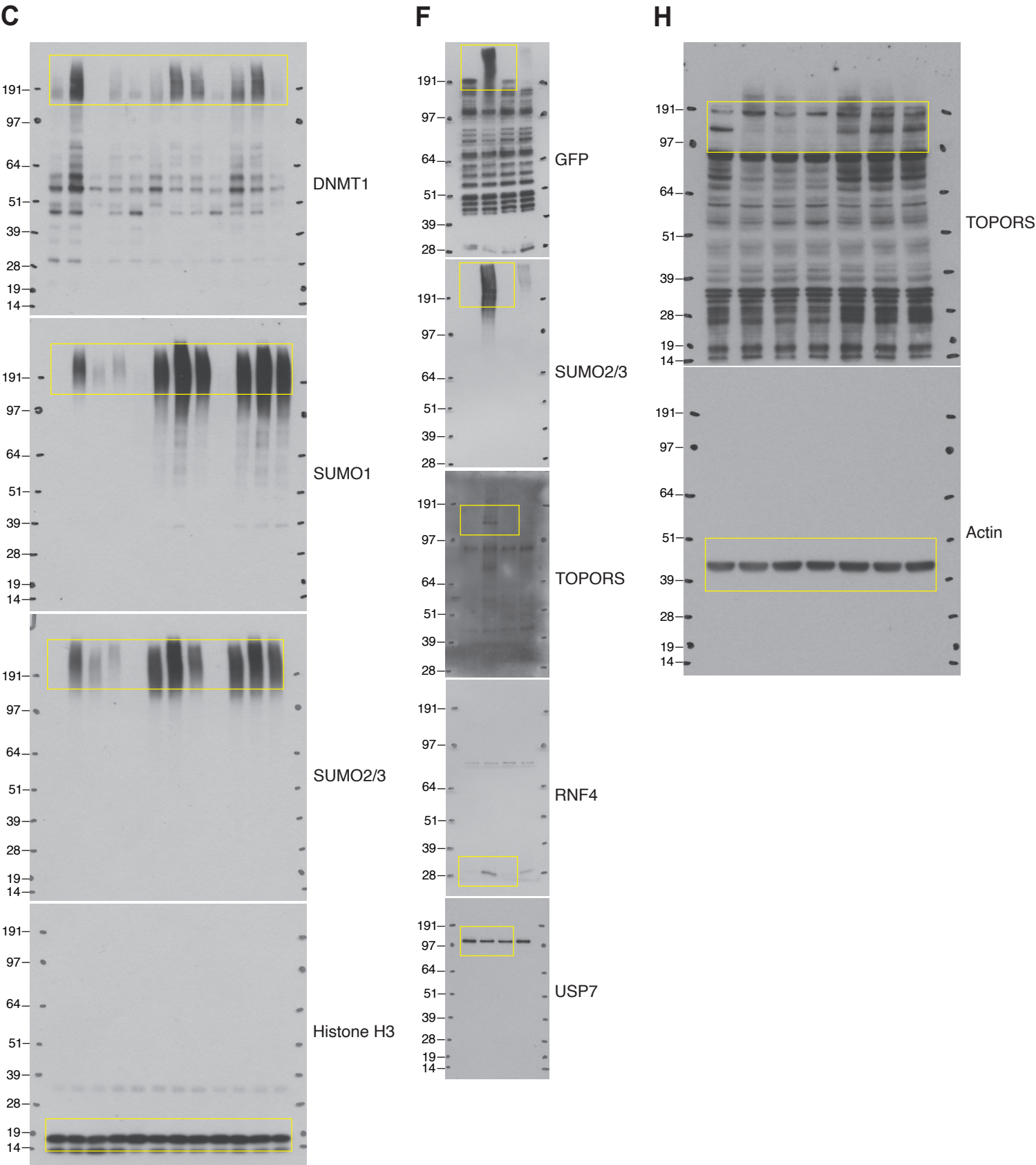

Supplement: Supplementary file 6 — Unprocessed western blots [file 41594_2024_1294_MOESM6_ESM.pdf]

Source Data Figure 2 - Uncropped scans

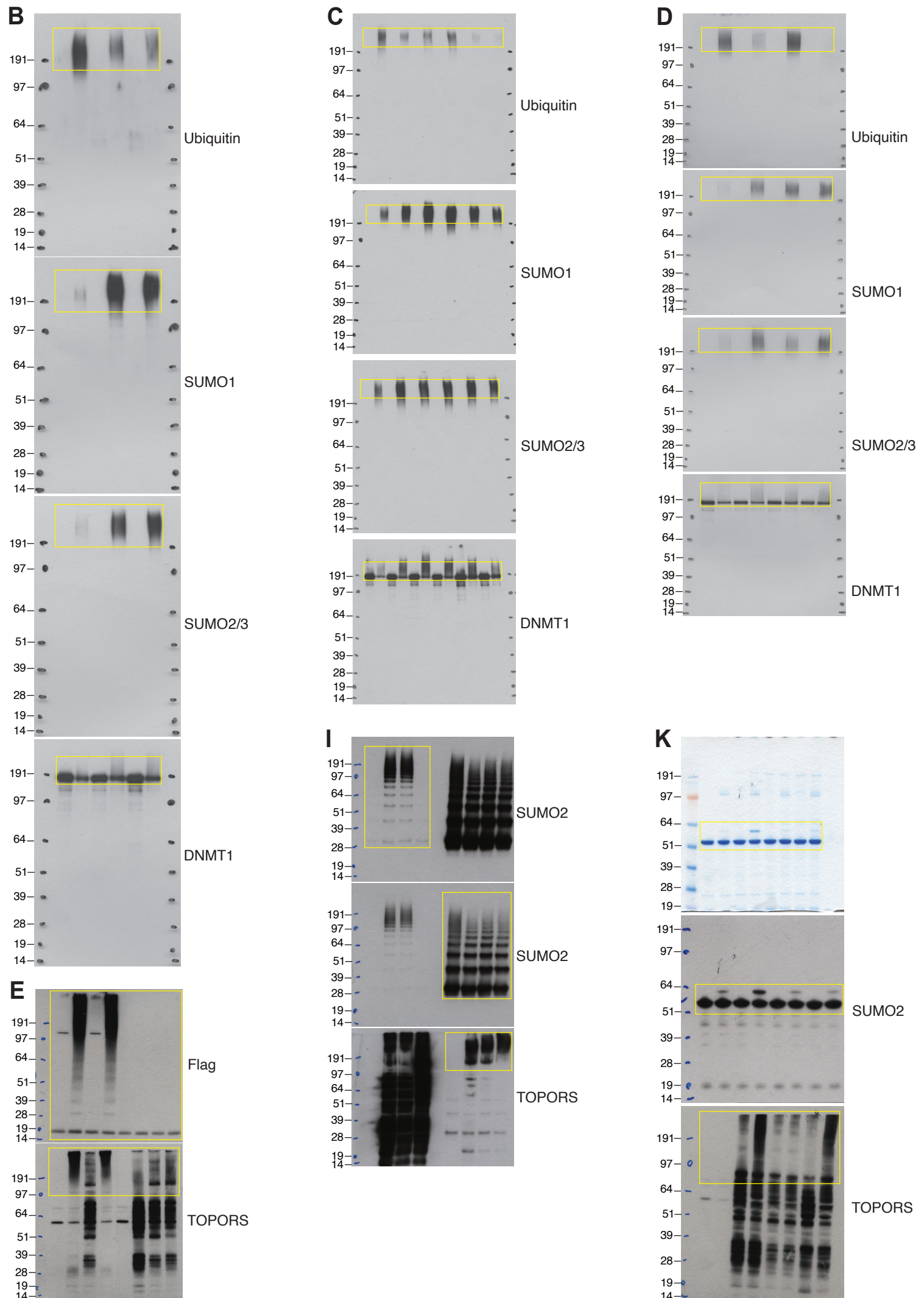

Supplement: Supplementary file 8 — Unprocessed western blots [file 41594_2024_1294_MOESM8_ESM.pdf]

Source Data Figure 3 - Uncropped scans

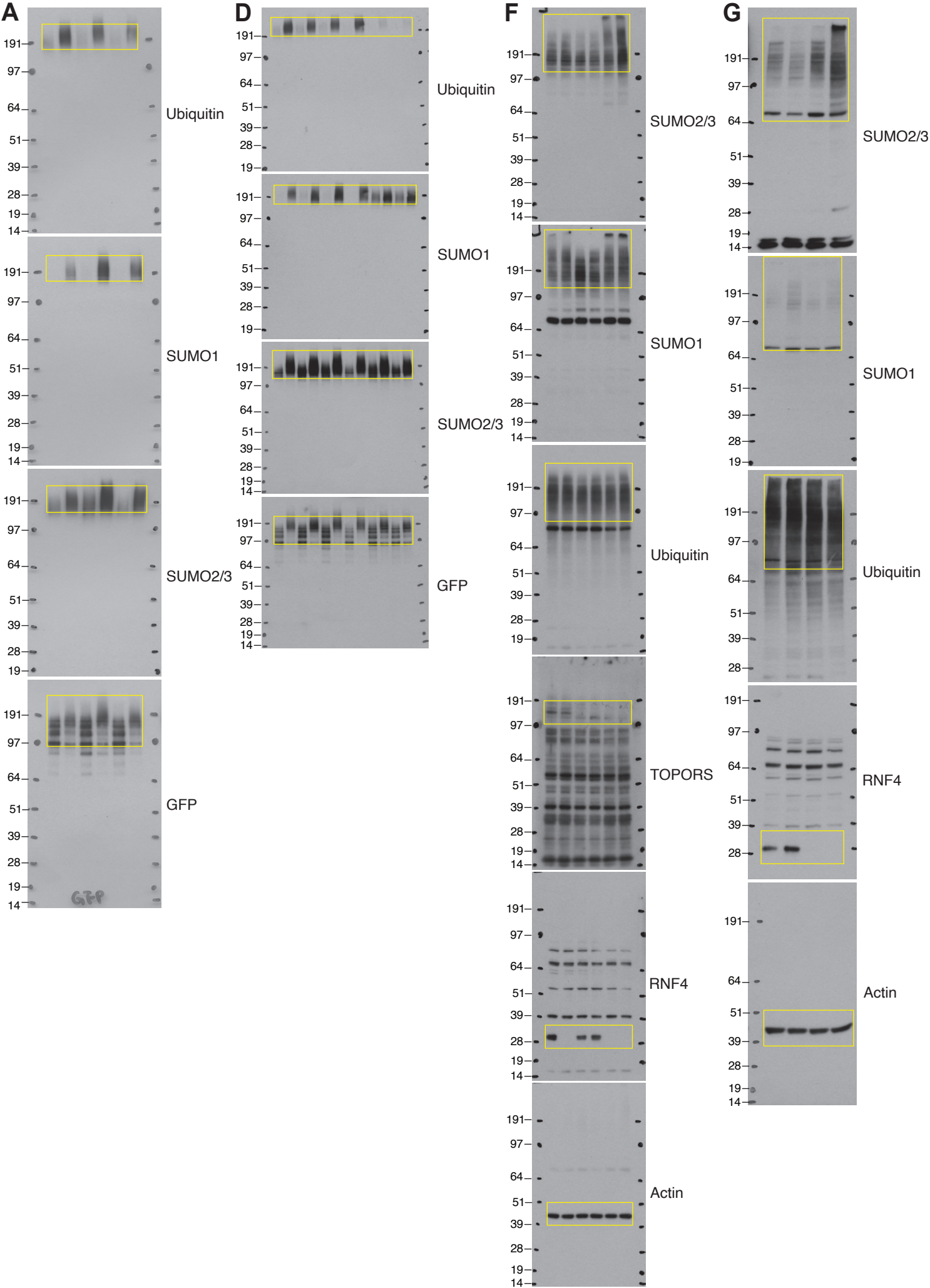

Supplement: Supplementary file 10 — Unprocessed western blots [file 41594_2024_1294_MOESM10_ESM.pdf]

Source Data Figure 4 - Uncropped scans

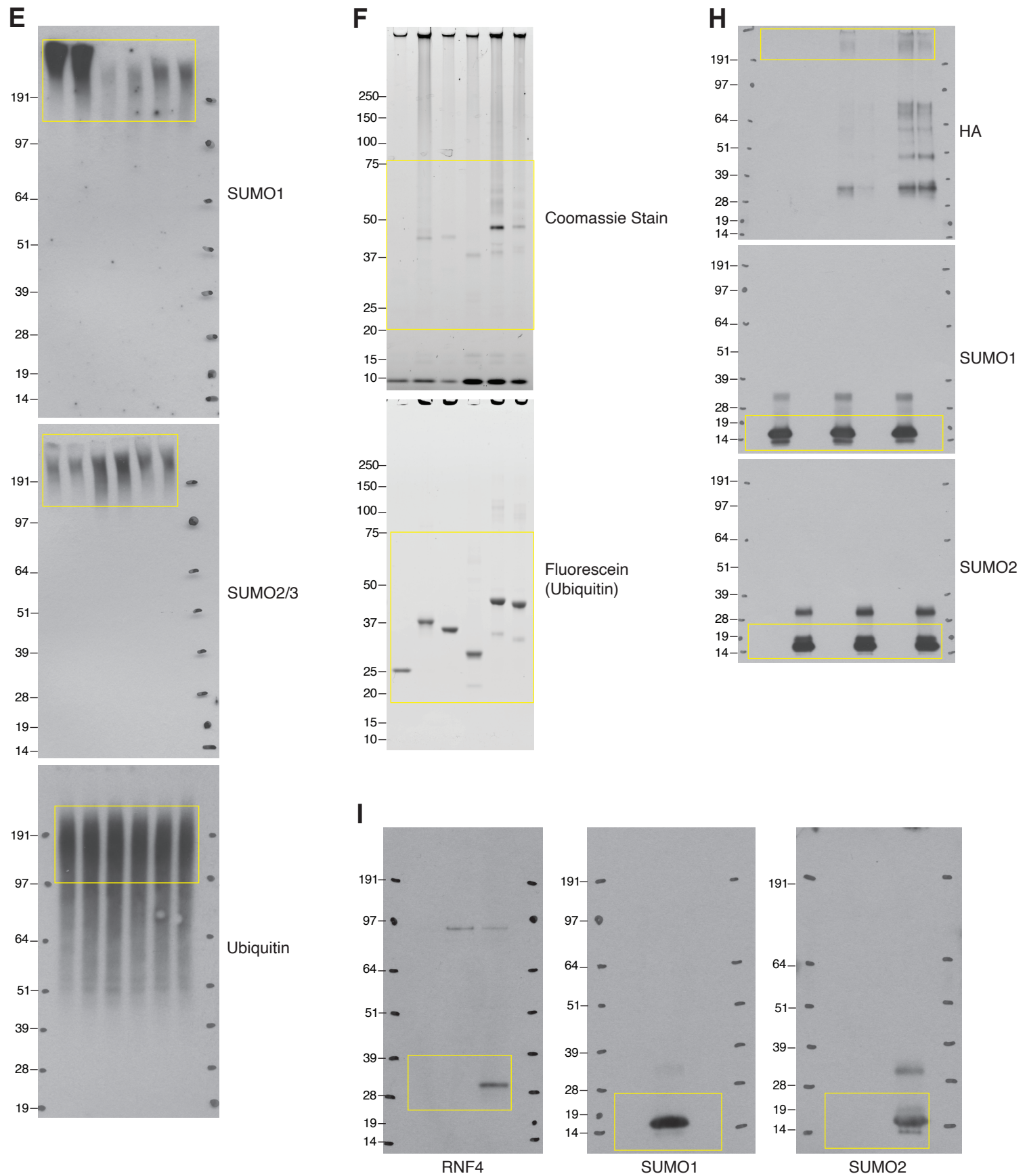

Supplement: Supplementary file 12 — Unprocessed western blots [file 41594_2024_1294_MOESM12_ESM.pdf]

Source Data Figure 5 - Uncropped scans

F

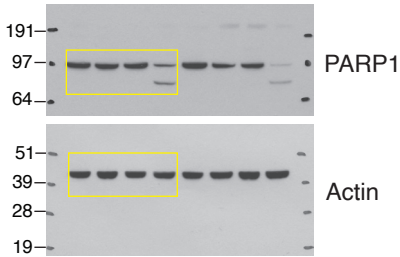

I

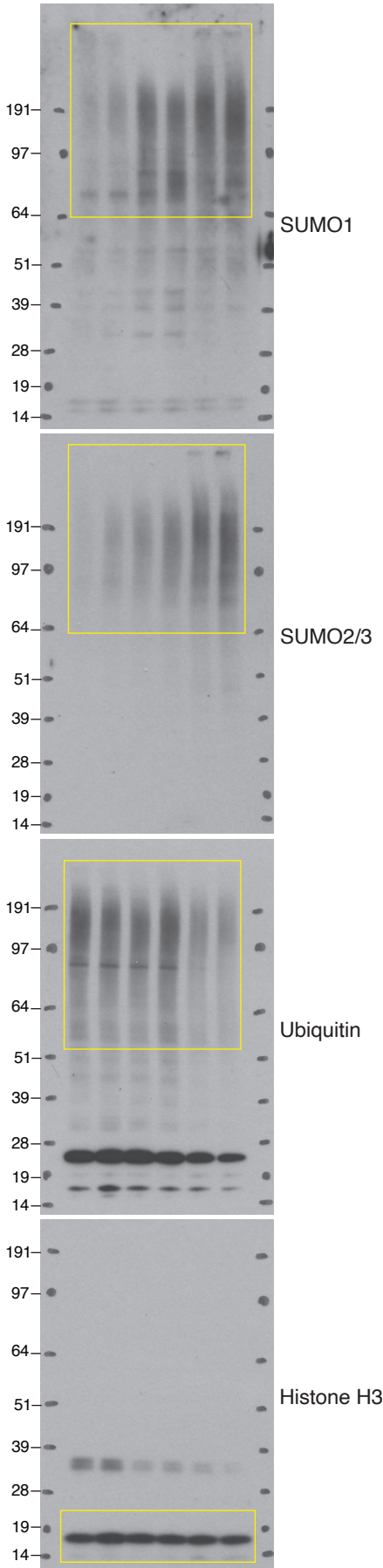

Supplement: Supplementary file 14 — Unprocessed western blots [file 41594_2024_1294_MOESM14_ESM.pdf]

# Source Data Extended Data Figure 1 - Uncropped scans

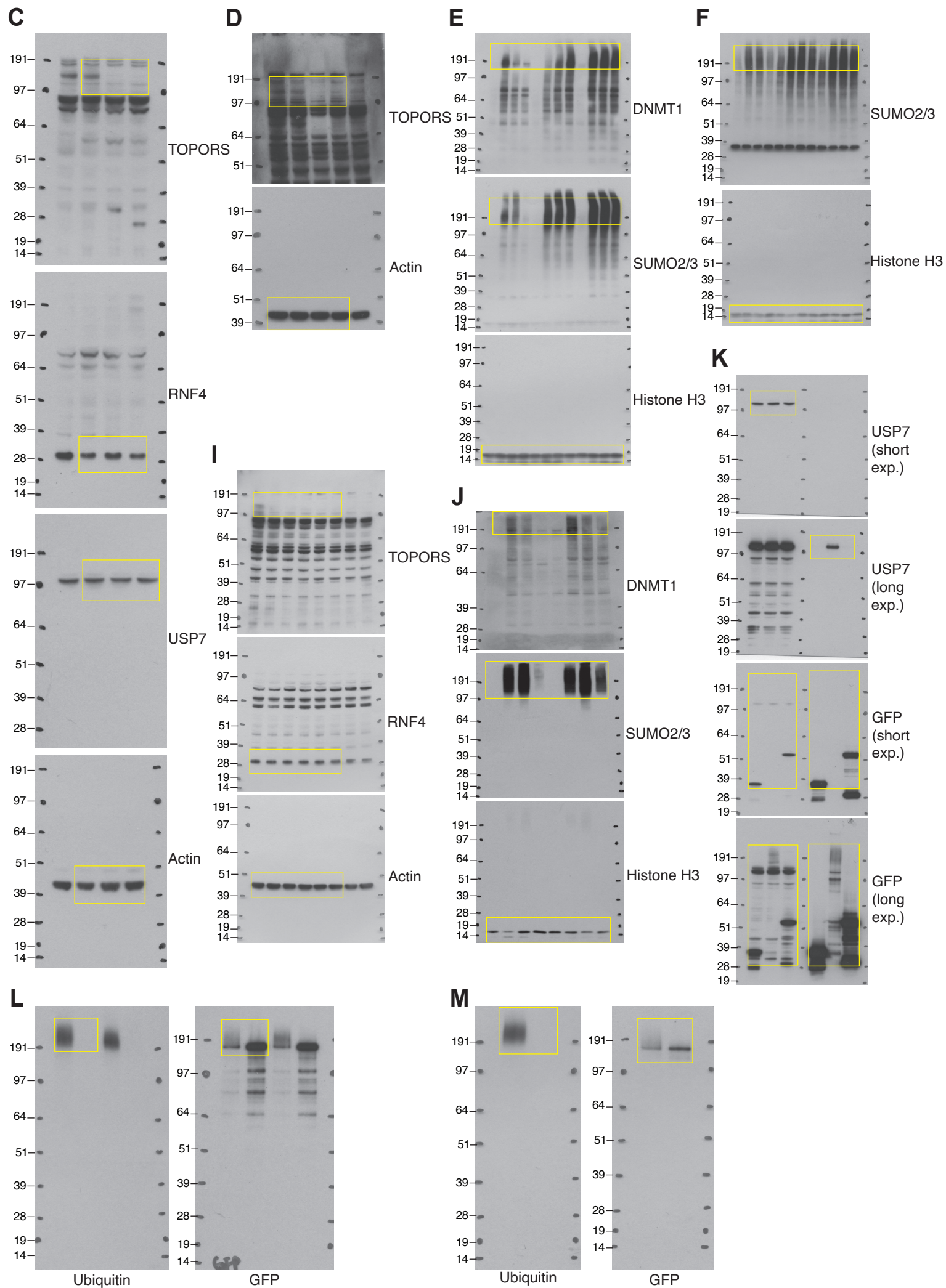

Supplement: Supplementary file 16 — Unprocessed western blots [file 41594_2024_1294_MOESM16_ESM.pdf]

Source Data Extended Data Figure 2 - Uncropped scans

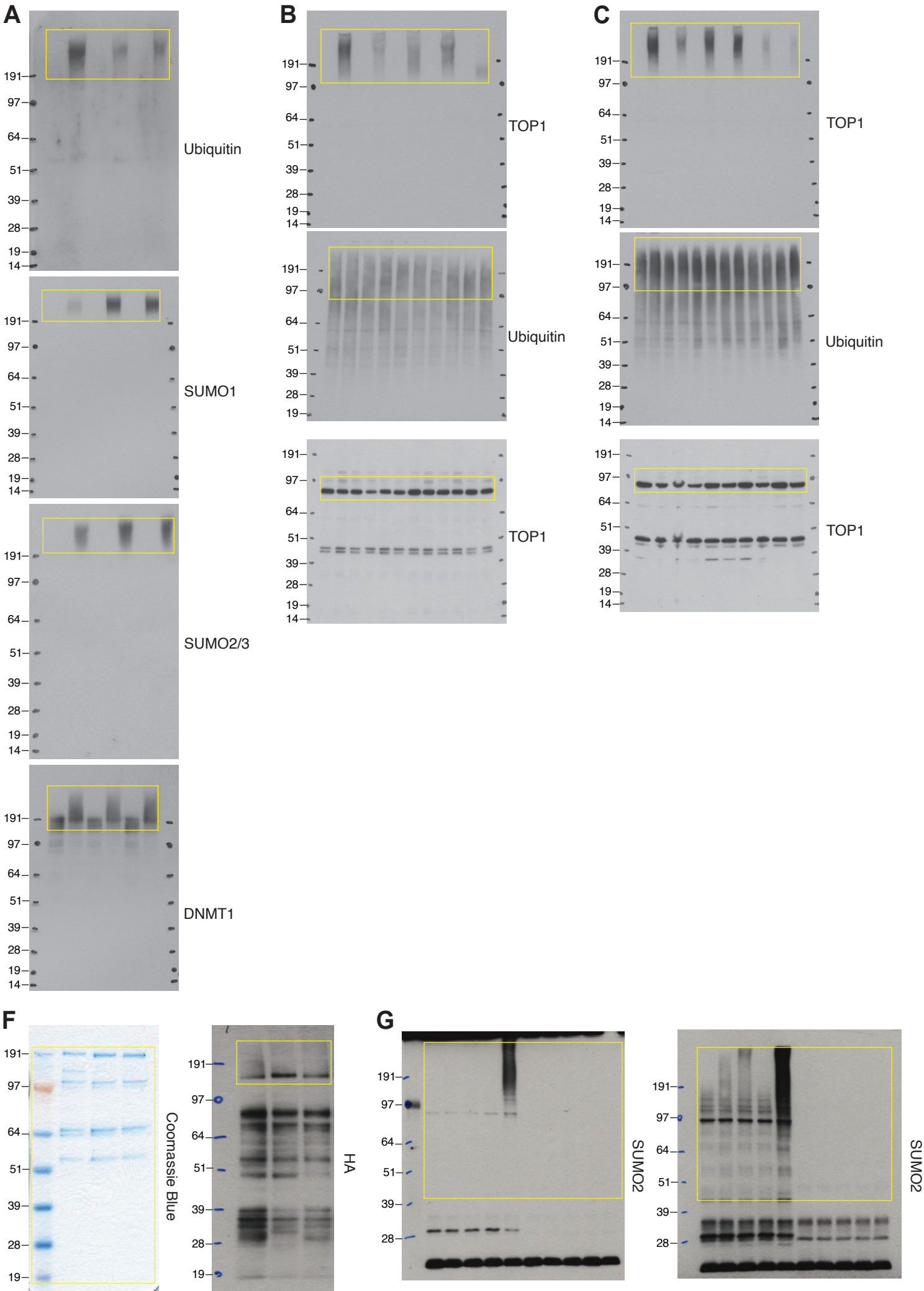

Supplement: Supplementary file 18 — Unprocessed western blots [file 41594_2024_1294_MOESM18_ESM.pdf]

Source Data Extended Data Figure 3 - Uncropped scans

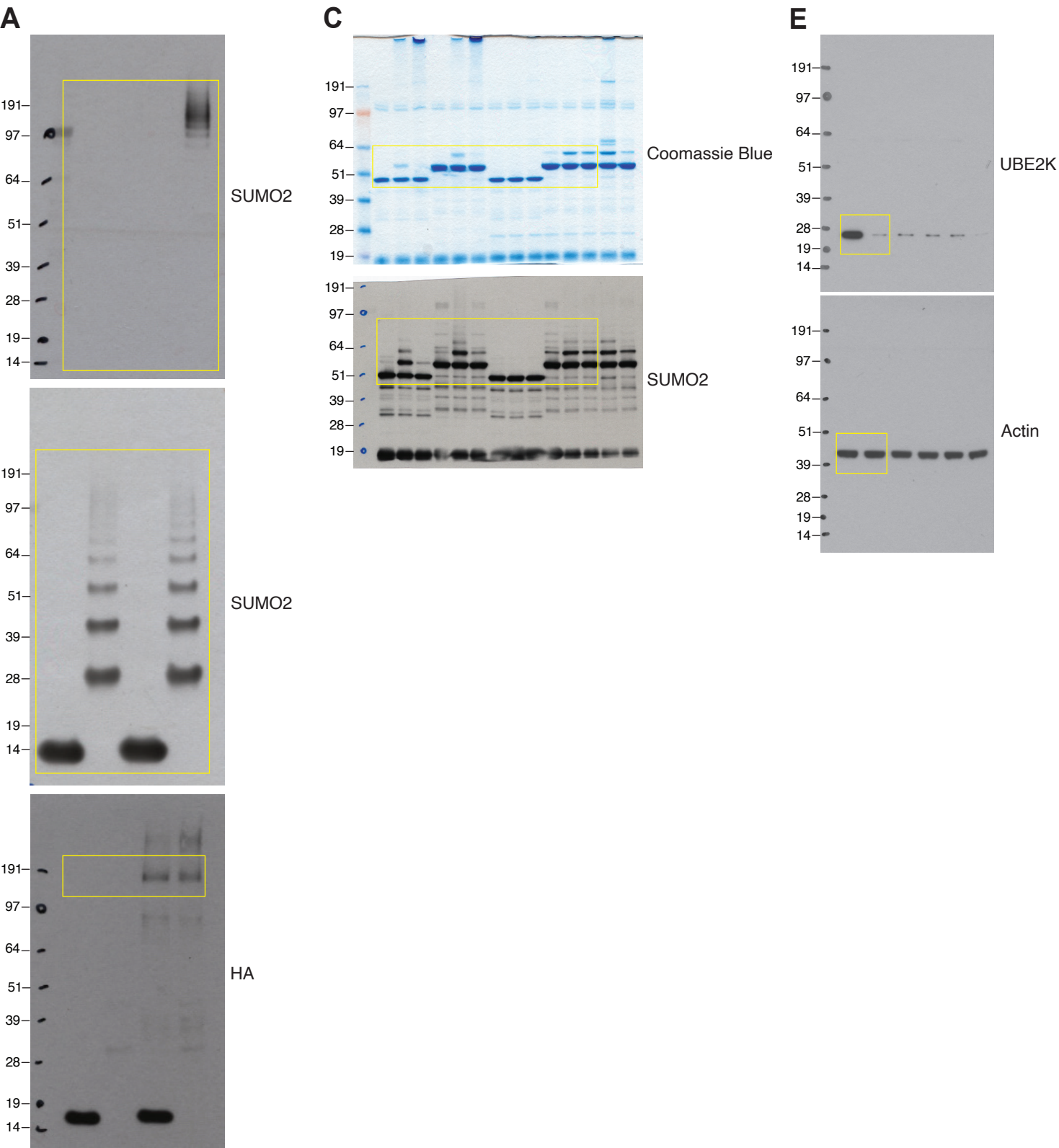

Supplement: Supplementary file 20 — Unprocessed western blots [file 41594_2024_1294_MOESM20_ESM.pdf]

A

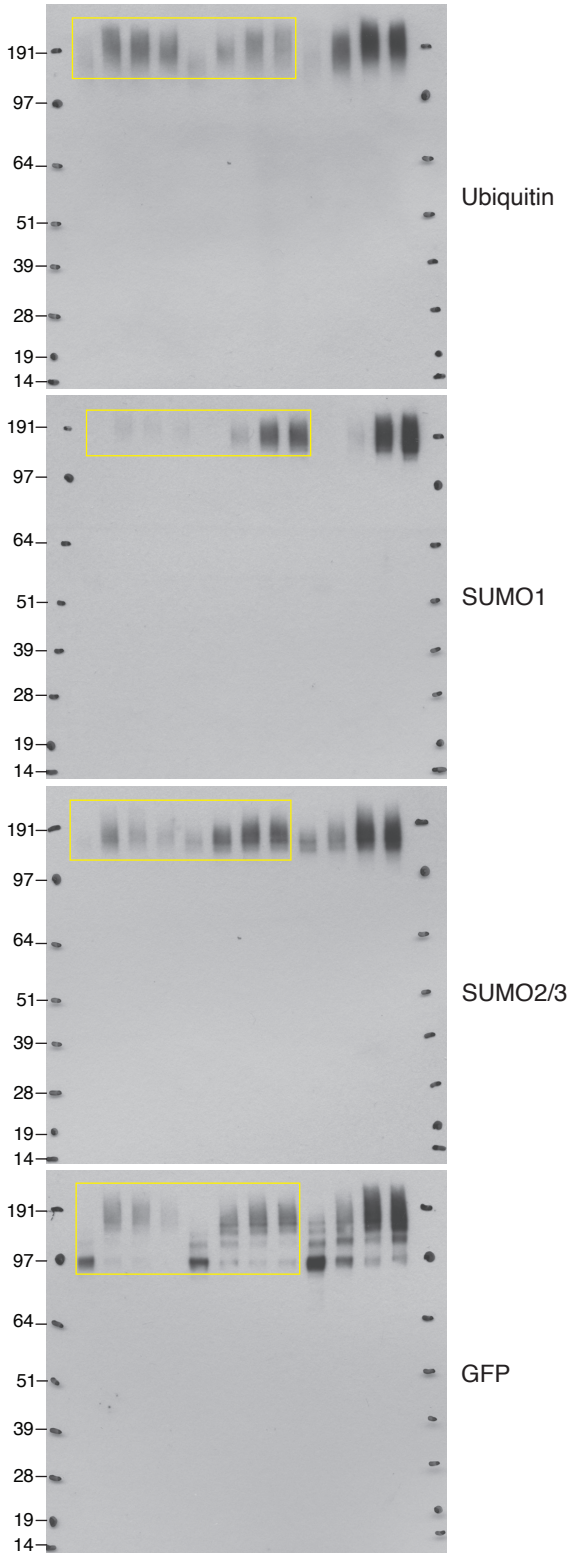

Supplement: Supplementary file 22 — Unprocessed western blots [file 41594_2024_1294_MOESM22_ESM.pdf]

Source Data Extended Data Figure 5 - Uncropped scans

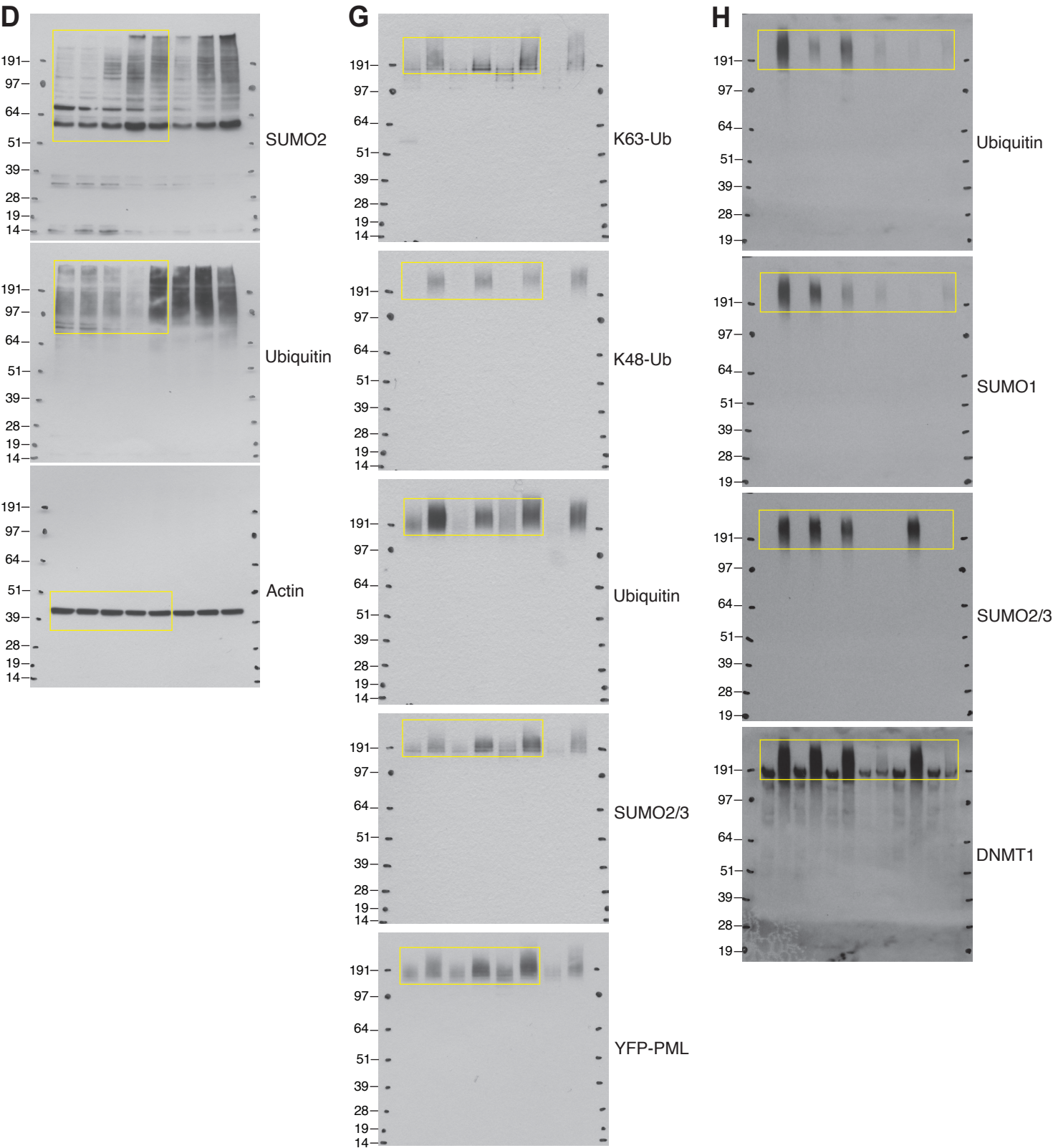

Supplement: Supplementary file 24 — Unprocessed western blots [file 41594_2024_1294_MOESM24_ESM.pdf]

Source Data Extended Data Figure 6 - Uncropped scans

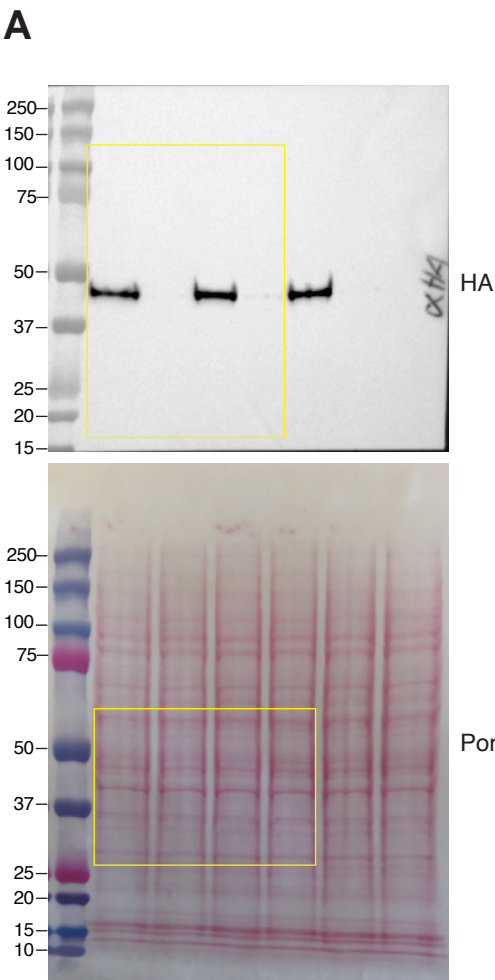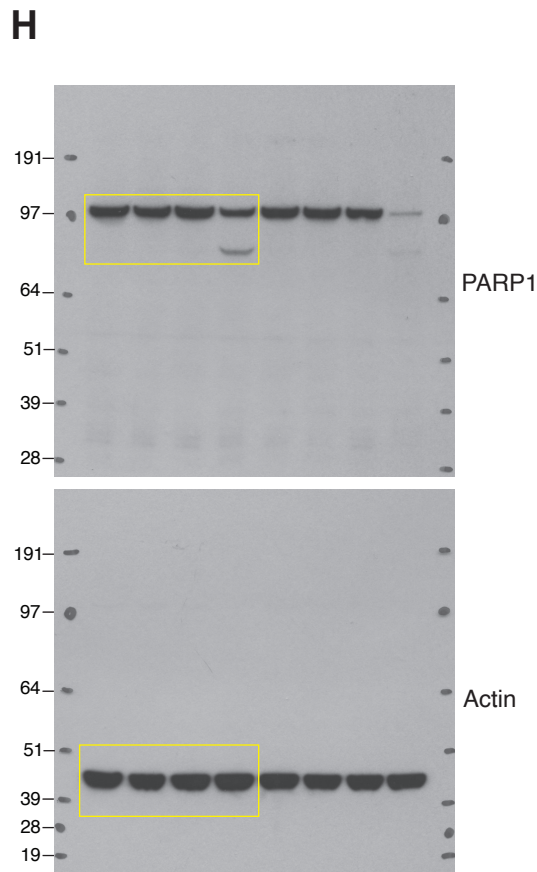

Supplement: Supplementary file 26 — Unprocessed western blots [file 41594_2024_1294_MOESM26_ESM.pdf]
